# Patterns of care for anal cancer in the United States - a comparison between academic and community cancer centers

**DOI:** 10.1186/s12885-018-4488-1

**Published:** 2018-05-16

**Authors:** Victor E. Pricolo, Matteo Bonvini, Carlo F. Abelli

**Affiliations:** 1 Southcoast Health, New Bedford, MA USA; 2 Alpert Medical School of Brown University, Providence, RI USA; 3 Harvard University, Cambridge, MA USA; 4 Yale University, New Haven, CT USA; 5 Department of Surgery, Southcoast Health, 300B Faunce Corner Road, No., Dartmouth, MA 02747 USA

**Keywords:** Anal cancer, HPV related cancer, Squamous cell carcinoma of the anus, NCDB, NCCN guidelines

## Abstract

**Background:**

Management of squamous cell carcinoma of the anus (SCCA) is becoming more relevant, as its incidence increases. The purpose of this study was to investigate possible differences in patient population and care delivery for SCCA between academic and community cancer programs in the United States.

**Methods:**

A review of available data from the American College of Surgeons Committee on Cancer National Cancer DataBase focused on gender, age, race, type of health insurance, comorbidity score, distance traveled for care, stage at diagnosis, and therapy utilization (surgery, chemotherapy, and radiation therapy) as first course of treatment (FCT). The analysis included 38,766 patients treated for SCCA. Of them, 14,422 patients received treatment at Academic Cancer Programs (ACPs), while 24,344 were treated at Community Cancer Programs (CCPs) between the years 2003 and 2013.

**Results:**

Over the 11-year study period, ACPs had significantly more male patients, of younger age, a greater non-white race population, with more Medicaid or no insurance coverage, who traveled farther for cancer center care (*p* < 0.001). There was no difference between ACPs and CCPs with respect to Charlson co-morbidity score and stage of SCCA at diagnosis. For stage 0 patients, use of chemotherapy was 8% for ACPs, 9% for CCPs, and use of radiotherapy was 10% for ACPs and 14% for CCPs. The incidence of stage unknown was identical at both ACPs and CCPs (11.5%). CCPs had a greater overall utilization of radiation therapy as FCT for stage 0, I, II and IV patients (*p* < 0.001).

**Conclusions:**

Our study indicates that gender, demographic and socio-economic differences exist in the patient population with SCCA accessing different cancer programs in the US. The high incidence of stage unknown patients reflects ongoing challenges in the pre-treatment phase. A significant percentage of stage 0 patients received systemic chemotherapy and/or radiotherapy, rather than surgery alone. Despite comparable stage at diagnosis and comorbidity scores between ACPs and CCPs, there appear to be variations in treatment choices, especially with the use of radiotherapy, with associated cost and toxicity risks. Further analysis and monitoring of SCCA management in the US may lead to improved compliance with NCCN guidelines.

## Background

The incidence of anal cancer has been steadily increasing in the US for approximately four decades, with an estimate by the American Cancer Society of 8080 new cases and 1080 deaths in 2016 [1, 2]. This trend has been attributed to the increased prevalence of anal human papillomavirus (HPV) infection in both men and women [3, 4], despite advances in diagnostic modalities and treatment options [5, 6]. The impact of the HPV vaccine for primary prevention may not effect a change of incidence of SCCA in the US for another two or three decades. However, its potential role as adjuvant therapy of anal cancer appears promising [7].

Anal cancer data collected in the National Cancer DataBase (NCDB) include both primary and metastatic tumors, for a total of 94 different histologic types, including melanomas, sarcomas, neuroendocrine tumors, and others. Our study focused only on the most common histologic type and its variants, squamous cell carcinoma of the anus (SCCA), which has been reported to account for about 90% of all cases [6].

The recommendations for care of patients with SCCA are outlined in the National Comprehensive Cancer Network (NCCN) guidelines [8]. Management of SCCA represents an example of coordinated multidisciplinary involvement, often including surgery, chemotherapy and radiotherapy, in order to provide accurate diagnosis, appropriate treatment and reliable survivorship plan. Such care delivery should be best accomplished within the organized and coordinated structure of an ACS CoC accredited Cancer Program. There are nine different categories of Cancer Programs. However, approximately 84% of patients are cared for at either Academic Cancer Programs (ACPs) or Community Cancer Programs (CCPs), the latter having the designation of “comprehensive” if they exceed 500 newly diagnosed cases per year [9]. The remaining 16% of cases include Integrated Network, Veterans Affairs, NCI- designated, Pediatric, and other programs. In order to have comparable sample sizes and civilian population, we chose to limit our analysis to ACPs and CCPs.

Multiple studies have observed differences in patterns of care for a variety of cancer sites (e.g. breast, thyroid) at different types of cancer centers, which have evolved over time [10, 11]. In this study we obtained available data from the NCDB on variables included in the management of SCCA in the United States to compare patient demographics and care delivery between ACPs and CCPs.

## Methods

The NCDB was established in 1989 as a nationwide, facility-based, comprehensive clinical surveillance resource oncology data set that currently captures information on approximately 70% of all newly diagnosed malignancies annually in the US. The NCDB is a joint project of the American Cancer Society and the Commission on Cancer of the American College of Surgeons, dedicated to the evaluation, management and surveillance of cancer patients in the US. The American College of Surgeons has executed a Business Associate Agreement that includes a data use agreement with each of its Commission on Cancer accredited hospitals. The database is populated by information entered by certified tumor registrars (CTR) from CoC accredited cancer centers. All Community Cancer Programs (CCP), with over or under 500 new cases per year, populated one data set. Academic Cancer Programs (ACP) populated the other data set for comparison. NCI-designated cancer programs, which account for under 2% of total data, were not included in the analysis.

We accessed data sets on “cancer of the anus, anal canal and anorectum” from 2003 to 2013, but selected for analysis only cases listed in the database with a histologic diagnosis containing the words “squamous cell carcinoma”, to ensure that our research focused on a homogeneous patient population. The staging system used was consistent with the AJCC Staging Manual 6th edition for data between 2003 and 2009, and the 7th edition from 2010 and 2013.

The NCDB web pages were exported to an Excel format and subsequently converted to a comma-separated value (CSV) file, which was processed through a custom script to generate results for analysis.

We obtained data on incidence variations over the 11-year study period.

Patient demographics such as gender, age, race, type of health insurance, Charlson comorbidity score, distance traveled for care, and stage at diagnosis were also collected. Finally, we extracted information on utilization of different therapeutic modalities, alone or in combination, as first course of treatment (FCT) for all reported stages of SCCA at initial diagnosis. The definition of FCT for “surgery” does not signify a diagnostic biopsy, but includes use of either local excision or radical resection. Similarly, the definitions of “chemotherapy” and “radiotherapy” as FCT, used alone or in combination, include utilization of different agents and dosages only as planned components of initial treatment, not for treatment failures or recurrences.

Subgroup comparisons of variables among different patient populations were performed using univariate analysis with the two-tailed, two-proportion z-test, and chi-square test. The Holm-Bonferroni method was then used to control the family-wise error rate and generate adjusted *p*-values. Statistical analyses were performed using R-software, version 3.2.2. All statistical test were two-sided, with statistical significance at *p* < 0.05.

## Results

From 2003 to 2013, a total of 38,766 cases of squamous cell carcinoma of the anus were identified, of which 14,422 were managed at ACPs, and 24,344 at CCPs.

The results of our analysis of patient characteristics between ACPs and CCPs are reported in Table 1.

**Table 1:** Comparison of demographics and stage at diagnosis for SCCA patients treated at ACPs and CCPs. Percentage values in parentheses

	ACP	CCP	P-value
	*n* = 14,422	*n* = 24,344	
Gender			
Male	6399 (44.4)	8831 (36.3)	< 0.0001*
Female	8023 (55.6)	15,513 (63.7)	
Age			
< 60	8787 (60.9)	12,516 (51.4)	
> 60	5635 (39.1)	11,828 (48.6)	< 0.0001*
Race			
White	10,553 (73.2)	20,939 (86)	
Non-white	3869 (26.8)	3405 (14)	< 0.0001*
Insurance			
P	5872 (41)	10,380 (42.6)	0.0002*
M	4513 (31)	9286 (38.1)	< 0.0001*
O	4037 (28)	4678 (19.2)	< 0.0001*
CC score			
0	11,392 (79)	19,370 (79.6)	0.1738
1–2	3030 (21)	4974 (20.4)	0.1787
Distance traveled			
< 10 miles	5842 (40.5)	10,952 (45)	< 0.0001*
> 25 miles	4341 (30.1)	5555 (22.8)	< 0.0001*
Stage			
0-I	3812 (26.4)	6629 (27.2)	0.0873
II-IV	8954 (62.1)	14,919 (61.3)	0.1164
Unknown	1656 (11.5)	2796 (11.6)	0.2133

With respect to gender, the male/female ratio was greater at ACPs: M = 6399 (44.4%)/F = 8023 (55.6%) than at CCPs: M = 8831 (36.3%)/F = 15,513 (63.7%) (*p* < 0.0001).

Age under 60 years was more frequent at ACPs: 8787 (60.9%) than CCPs: 12516 (51.4%), and age over 60 years was less frequent at ACPs: 5635 (39.1%) than CCPs: 11828 (48.6%) (*p* < 0.0001).

Race was more commonly non-white at ACPs: 3869 (26.8%) than CCPs: 3405 (14%), and less commonly white at ACPs: 10553 (73.2%) than CCPs: 20939 (86%) (*p* < 0.0001).

Insurance status was grouped as Private/Managed (P), Medicare (M), and Medicaid/Not insured, unknown and others (O). ACPs had fewer patients with P: 5872 (41%) than CCPs:10380 (42.6%) (*p* = 0.0002). ACPs had fewer patients with M: 4513 (31%) than CCPs: 9286 (38.1%) (*p* < 0.0001), and ACPs had more patients with O: 4037 (28%) than CCP: 4678 (19.2%) (*p* < 0.0001).

Charlson comorbidity score (CC) was not significantly different between patients treated at ACPs and CCPs.

Distance traveled to access cancer center care was less often less than 10 miles for patients treated at ACPs (40.5%) than for patients treated at CCPs (45%) (*p* < 0.0001); and greater than 25 miles more often for patients who received care at ACPs (30.1%) than at CCPs (22.8) (p < 0.0001).

Stage at diagnosis was not significantly different between patients treated at ACPs and CCPs.

The results of our analysis of type of therapy, by stage of SCCA at diagnosis, between ACPs and CCPs are reported in Table 2.

**Table 2:** Comparison of different treatment modalities by stage for SCCA patients treated at ACPs and CCPs. Percentage values in parentheses

	ACP	CCP	P-value
Stage 0	*n* = 1351	*n* = 2227	
S only	1016	1660	
R only	19	32	
C only	12	10	
S + R	29	85	
S + C	9	10	
R + C	51	105	
S + R + C	35	84	
Other	180	241	
S overall	1089 (80.6)	1839 (82.5)	0.1506
R overall	134 (9.9)	306 (13.7)	0.0009*
C overall	107 (7.9)	209 (9.4)	0.1510
Stage I	*n* = 2461	*n* = 4402	
S only	655	990	
R only	60	108	
C only	22	28	
S + R	109	208	
S + C	27	41	
R + C	716	1433	
S + R + C	758	1437	
Other	114	157	
S overall	1459 (62.9)	2676 (60.8)	0.0834
R overall	1643 (66.8)	3186 (72.4)	< 0.0001*
C overall	1523 (61.9)	2939 (66.8)	< 0.0001*
Stage II	*n* = 4512	*n* = 8453	
S only	428	558	
R only	164	313	
C only	43	56	
S + R	101	203	
S + C	27	63	
R + C	2634	5052	
S + R + C	970	1950	
Other	145	258	
S overall	1526 (33.8)	2774 (32.8)	0.2554
R overall	3869 (85.7)	7518 (88.9)	< 0.0001*
C overall	3674 (81.4)	7121 (84.2)	< 0.0001*
Stage III	*n* = 3744	*n* = 5396	
S only	82	108	
R only	132	193	
C only	66	61	
S + R	39	52	
S + C	21	32	
R + C	2684	3898	
S + R + C	588	859	
Other	132	193	
S overall	730 (19.5)	1051 (19.5)	0.1443
R overall	3443 (92)	5002 (92.6)	0.2102
C overall	3359 (89.7)	4850 (89.9)	0.1849
Stage IV	*n* = 698	*n* = 1070	
S only	17	31	
R only	43	69	
C only	117	126	
S + R	6	13	
S + C	24	23	
R + C	342	578	
S + R + C	66	105	
Other	83	125	
S overall	113 (16.2)	172 (16.1)	0.9522
R overall	457 (65.5)	765 (71.5)	0.0074*
C overall	549 (78.7)	832 (77.8)	0.6527
Stage unknown	*n* = 1656	*n* = 2796	
S only	371	521	
R only	86	137	
C only	45	88	
S + R	37	50	
S + C	20	30	
R + C	535	1008	
S + R + C	280	414	
Other	282	548	
S overall	708 (42.8)	1015 (36.3)	< 0.0001*
R overall	938 (56.6)	1609 (57.5)	0.5769
C overall	880 (53.1)	1540 (55.1)	0.2209

More patients in stage 0 received radiotherapy at CCPs (13.7%) than at ACPs (9.9%) (*p* = 0.0009). In stage I patients, there was a greater overall utilization of radiotherapy at CCPs (72.4%) than ACPs (66.8%) (*p* < 0.0001), as well as chemotherapy: CCPs (66.8%) vs ACPs (61.9%) (*p* < 0.0001). A similar treatment pattern difference was present in stage II patients for overall use of radiotherapy: CCPs (88.9%) vs. ACPs (85.7%) (*p* < 0.0001), and for overall use of chemotherapy: CCPs (84.2%) vs. ACPs (81.4%) (p < 0.0001). Also, in stage IV patients, CCPs showed a greater use of radiotherapy (71.5%) than ACPs (65.5%) (*p* = 0.0074). In stage unknown patients, there was a greater use of surgery at ACPs (42.8%) than at CCPs (36.3%) (*p* < 0.0001).

## Discussion

Squamous cell carcinoma of the anus remains a relatively rare cancer, but its incidence has now increased to 2.6% of all new cancer cases of the digestive tract diagnosed in the US in 2016 [12]. Any single center is unlikely to have a large institutional experience with SCCA; therefore, a large national database review offers the most meaningful approach for analyzing data on this malignancy [13, 14].

Risk factors for invasive SCCA are similar to those of cervical cancer, with intraepithelial neoplasia being identified as the precursor lesion. Most studies have detected high-risk human papilloma virus (HPV), predominantly HPV-16, in over 80% of cases of SCCA [15, 16].

Clinical presentation, histologic confirmation by biopsy, diagnostic workup, and clinical staging principles are well outlined in the most recent edition of the NCCN practice guidelines [8].

In our comparison of patterns of care at ACPs versus CCPs in the US, there was an identically high incidence of stage unknown of 11.5% (Table 2). Such finding suggests ongoing problems in accurate staging of this type of neoplasm.

Our study found that patients that received care at ACPs, when compared to patients treated at CCPs, were more often of male gender, more often younger than age 60, and traveled over 25 miles more frequently to access cancer center care. Patients treated at ACPs were also less often of white race and more rarely carried health insurance. Although our study does not intend to prove a causal relationship, it does draw attention to the importance of considering socio-demographic factors in evaluating availability and utilization of therapeutic resources for cancer care in the US.

In our review, the patient population treated at ACPs and CCPs was homogeneous with respect to stage at diagnosis as well as presence of comorbidities that might affect treatment choices.

Stage 0 patients, who under most circumstances should be best managed surgically, received chemotherapy in 8% of cases for ACPs, 9% for CCPs, and radiotherapy in 10% of cases for ACPs and 14% for CCPs. Possible explanations for such findings may include lack of awareness of staging system, limited involvement of a colorectal surgical specialist in the diagnostic and staging phase, possibly as a result of the relative rarity of this neoplasm.

Patients treated at CCPs received radiation therapy (R), alone or in combination with other treatment modalities, for stage 0, stage I, stage II and stage IV disease significantly more often than patients treated at ACPs (Table 2).

Patients treated at CCPs also received chemotherapy more often than patients treated at ACPs in stages I and II. It appears that, in a significant percentage of patients, chemotherapy and/or radiotherapy were used as FCT, regardless of stage. Particularly for patients in stages 0 or unknown, such therapeutic practices may carry significant side effects as well as “financial toxicity”, with the cost of radiation therapy for anal cancer often exceeding $55,000, including treatment planning, simulation, and professional charges [17, 18].

## Conclusions

This study of patients with SCCA in the NCDB found that there are challenges with respect to accurate staging of SCCA cases, with a high percentage of patients being managed with “stage unknown”. SCCA has become an increasingly common cancer that poses unique challenges in prevention, diagnosis, accurate staging, therapy and survivorship. There are socio-demographic variations as well differences in care delivery between ACPs and CCPs in the US. Limitations of this work include inability to access data on squamous cell carcinoma of the anal margin cases, as they are currently not being entered in the NCDB. Additionally, our study did not address different surgical procedures, different chemotherapeutic agents, or radiation doses utilized as FCT. The purpose of this work was primarily to draw attention to variations in care strategies in management of a cancer whose incidence will continue to increase for the next decade or two, until HPV vaccine recipients in the US reach their 50s. A greater involvement of qualified surgeons in the care team, additional scientific investigations, improved awareness and closer motoring of guidelines concordant care should lead to quality improvement in the management of SCCA.

## Abbreviations

ACP: Academic cancer program
ACS: American College of Surgeons
C: Chemotherapy
CCP: Community cancer program
CoC: Commission on Cancer
CTR: Certified Tumor Registrar
FCT: First course of treatment
GCC: Guidelines concordant care
HIV: Human immunodeficiency virus
HPV: Human papillomavirus
NCCN: National Comprehensive Cancer Network
NCDB: National Cancer DataBase
R: Radiotherapy
S: Surgery
SCCA: Squamous cell carcinoma of the anus
US: United States of America
WHO: World Health Organization
